# Immune response profiling identifies autoantibodies specific to Moyamoya patients

**DOI:** 10.1186/1750-1172-8-45

**Published:** 2013-03-21

**Authors:** Tara K Sigdel, Lorelei D Shoemaker, Rong Chen, Li Li, Atul J Butte, Minnie M Sarwal, Gary K Steinberg

**Affiliations:** 1 California Pacific Medical Center Research Institute, San Francisco, CA, 94107, USA; 2 Department of Neurosurgery, Stanford University School of Medicine, Stanford, CA, 94305, USA; 3 Stanford Institute for Neuro-Innovation & Translational Neurosciences, Stanford University, Stanford, CA, 94305, USA; 4 Department of Pediatrics, Stanford University School of Medicine, Stanford, CA, 94305, USA; 5 Pediatrics and Transplant Nephrology, California Pacific Medical Center Research Institute, 475 Brannan Street, San Francisco, CA, 94107, USA; 6 Stanford Stroke Center, 300 Pasteur Drive, R281, Stanford, CA, 94305-5327, USA

**Keywords:** Autoantibodies, Cerebrovascular disease, Moyamoya, Protein microarray

## Abstract

**Background:**

Moyamoya Disease is a rare, devastating cerebrovascular disorder characterized by stenosis/occlusion of supraclinoid internal carotid arteries and development of fragile collateral vessels. Moyamoya Disease is typically diagnosed by angiography after clinical presentation of cerebral hemorrhage or ischemia. Despite unclear etiology, previous reports suggest there may be an immunological component.

**Methods:**

To explore the role of autoimmunity in moyamoya disease, we used high-density protein arrays to profile IgG autoantibodies from the sera of angiographically-diagnosed Moyamoya Disease patients and compared these to healthy controls. Protein array data analysis followed by bioinformatics analysis yielded a number of auto-antibodies which were further validated by ELISA for an independent group of MMD patients (n = 59) and control patients with other cerebrovascular diseases including carotid occlusion, carotid stenosis and arteriovenous malformation.

**Results:**

We identified 165 significantly (p < 0.05) elevated autoantibodies in Moyamoya Disease, including those against CAMK2A, CD79A and EFNA3. Pathway analysis associated these autoantibodies with post-translational modification, neurological disease, inflammatory response, and DNA damage repair and maintenance. Using the novel functional interpolating single-nucleotide polymorphisms bioinformatics approach, we identified 6 Moyamoya Disease-associated autoantibodies against APP, GPS1, STRA13, CTNNB1, ROR1 and EDIL3. The expression of these 6 autoantibodies was validated by custom-designed reverse ELISAs for an independent group of Moyamoya Disease patients compared to patients with other cerebrovascular diseases.

**Conclusions:**

We report the first high-throughput analysis of autoantibodies in Moyamoya Disease, the results of which may provide valuable insight into the immune-related pathology of Moyamoya Disease and may potentially advance diagnostic clinical tools.

## Background

Moyamoya Disease (MMD) is a rare cerebrovascular disorder that involves progressive bilateral stenosis/occlusion of the supraclinoid internal carotid arteries (ICAs) as well as the proximal anterior and middle cerebral arteries (ACAs and MCAs) [1,2]. MMD is also characterized by the development of abnormal, fragile collateral blood vessels, usually from enlarged and proliferated lenticulostriate arteries [3]. Early diagnosis is difficult and is usually made angiographically after clinical presentation of cerebral ischemia (stroke or TIA) or hemorrhage, often well after damage to the brain has occurred. The etiology of MMD is currently unknown. Approximately 10–12% of MMD cases are clearly familial with an autosomal dominant inheritance pattern, and several MMD-associated loci have been identified, including 3p24.2-p26, 6q25, 8q23, 12p12, and 17q25.3 [1,4]. Mutations in individual genes have also been described, including TGFβ1 and PDGFRB [5], BRCC3 [6], and RNF213 [1,4,7]. There is also some evidence for active vascular remodeling in MMD collaterals, including proliferation of the smooth muscle cell layer in the intima of MMD vessels [1,4]. The expression levels of several proteins with known roles in angiogenesis have been described, including vascular endothelial growth factor (VEGF), basic fibroblast growth factor (bFGF) and transforming growth factor beta (TGF-β) [1,4]. There are also reports of increased intercellular adhesion molecule-1 (ICAM-1) and vascular cell adhesion molecule-1 (VCAM-1) in the cerebral spinal fluid (CSF) of MMD patients [8].

MMD is often described as Moyamoya Syndrome (MMS) when it is associated with other disorders, such as Majewski Osteodysplastic Primordial Dwarfism Type II (MOPD II), Down syndrome, Seckel syndrome, Neurofibromatosis and Graves’s disease, a known autoimmune disorder [9-11]. Although MMD is not traditionally considered an autoimmune disease, several groups have reported elevated levels of circulating autoantibodies (autoAbs), including anti-cardiolipin [12], anti-thyroid [13,14] and anti-α-fodrin [15]. In addition, a SEREX profile of CSF obtained from MMD patients identified 5 proteins against which autoAbs were expressed [16].

To comprehensively characterize the serum autoAb repertoire in MMD patients, our unbiased approach used a commercially-available high-density protein microarray platform to identify circulating serum autoAbs. These arrays have been useful for profiling disease-relevant autoAbs and have led to the identification of novel autoAbs in chronic renal [17], rheumatologic and autoimmune diseases [18]. Our study identified a number of MMD-associated autoAbs and the biological pathways and networks associated with them.

## Methods

### Patients and samples

The study comprised both a discovery and a validation phase and included 88 serum samples, 56 of which were from MMD patients. The discovery phase involved 10 samples from healthy normal controls (mean age 29.3 ± 13.1 yrs) and 10 samples from demographically-matched MMD patients (mean age 27.6 ± 10.7 yrs, 5 MMD patients collected at 2 time points for each patient: preoperative and 6 months postoperative). AutoAbs of interest in MMD were validated in an independent patient cohort of 68 independent samples—46 with MMD (mean age 40.7 ± 13.6 yrs) and 22 from matched control patients with other cerebrovascular diseases (CVDs) (mean age 49.1 ± 21.6 yrs) including carotid occlusion (n = 8), carotid stenosis (n = 5) and arteriovenous malformation (n = 9). MMD was radiographically defined in all cases, with the presence of characteristic unilateral or bilateral stenosis/occlusion of the internal carotid artery (including involvement of the anterior cerebral artery and the middle cerebral artery (MCA)) and the presence of classic small caliber collateral ‘moyamoya’ vessels. Our MMD and CVD cohort was compromised of 46% and 52% respectively, of patients with no other clinical condition other than cerebrovascular disease. Hypertension and hyperlipidemia were present in 24% and 40% of the MMD and CVD cohort respectively. No patients were clinically diagnosed with Sickle cell disease or neurofibromatosis, nor did any have a history of cranial x-irradiation; 2 MMD patients also had Down syndrome however. The demographic information is summarized in Table 1.

**Table 1:** Demographic data for control and MMD patients

	**Discovery set (20 sera)**			**Validation set (68 sera)**		
	**Protein arrays analysis**			**Cross sectional ELISA**		
	**Healthy control**	**MMD**		**CVD control**	**MMD**	
	**n = 10**	**n = 10**	**P value**	**n = 22**	**n = 46**	**P value**
**Gender: males (%)**	40	60	0.61	45	26	0.03
**Age (yr)**	29.6 ± 12.2	27.8 ± 10.7	0.73	49.1 ± 21.6	40.7 ± 13.6	0.10
**Ethnicity% (1/2/3/4) †**	30/60/10/0	20/80/0/0	0.66	59/36/0/5	54/28/9/9	0.45
**(MMD) Unilateral/bilateral**	na	0/5	na	na	24/22	na

MMD = moyamoya disease; CVD = cerebrovascular disease.

†1, Caucasian; 2, Asian; 3, Hispanic 4. Other.

Using standard methods for sera collection [17], 5.0 mL of blood from each patient was collected, and the serum was separated by centrifugation, aliquoted and immediately stored at −80°C until use. The samples were collected under consent at the Stanford Hospital and Clinics from 2006–2008. The study was approved by the Institutional Review Board of Stanford University.

### Immune response profiling using high-density protein arrays

The ProtoArray® Human Protein Microarray v4.0 (Invitrogen, Carlsbad, CA) was used to characterize IgG reactivity in MMD. V4.0 arrays contain 8268 recombinant human proteins expressed as N-terminal GST fusion proteins. We used our standard protocols as previously described for serum sample preparation, blocking, probing, drying, scanning, and data acquisition [19]. Briefly, ProtoArrays were blocked with 5.0 mL blocking buffer (100 mM sodium phosphate pH 7.4, 200 mM NaCl, 0.08% Triton X-100, 25% glycerol, 20 mM glutathione, 1.0 mM DTT, 1.0% casein) for 1 hour at 4°C. After the blocking step, 5.0 mL of diluted serum sample (1:150 in PBST buffer consisting of PBS, 1% casein and 0.1% Tween 20) was applied and incubated for 90 minutes at 4°C. The slides were washed 5 × 5 minutes with PBST buffer prior to application of secondary antibody (anti-human antibody-Alexa Fluor® 647 conjugate). The slides were incubated for 90 minutes at 4°C, washed for 5 × 5 minutes with PBST, dried and then scanned using the Axon GenePix 4000B Scanner (Molecular Devices, Sunnyvale, CA).

### Data extraction

Signal intensities per spot for each individual array were obtained using GenePix pro 6.0. We used Prospector Analyzer® 5.2 (Invitrogen Inc., Carlsbad, CA) to subtract the background, and to normalize and analyze GenePix Results (GPR) files for each array.

### Data analysis for MMD-specific autoAbs

We used a standard cut-off Z-Score of 3.0. Individual antigen reactivity was ranked based on Z-score above or below the mean signal for each array. Arrays from patients of a distinct clinical phenotype were analyzed as a group. Group analyses were made by comparing 2 sets of individual antibody levels for every antigen present on the array using M-statistics of the Prospector Analyzer® with the Robust-Linear-Normalization (RLM) method [20]. Differences in significance were displayed as ANOVA and Chebyshev’s Inequality p-Value (≤ 0.05 considered significant). For significant autoAbs in MMD, p-value was calculated with unpaired t-test.

To analyze MMD-associated pathways and major functions we used Ingenuity Pathway Analysis® (http://www.ingenuity.com) for Global Functional Analysis (GFA) and Global Canonical Pathways (GCP) Analysis, which produced a list of major pathways and functions with p-values.

### Bioinformatics prediction of MMD-associated autoAbs by functional interpolating SNPs (fitSNPs)

We used a novel data analysis method to identify MMD-specific autoAbs by speculating that those proteins would have a higher chance of being associated with proteins with a higher probability of having functionally-relevant single-nucleotide polymorphisms (SNPs). For this purpose we used a method that employs an integration of genetic and genomic data called functional interpolating SNPs (fitSNPs) [21]. First, we identified three loci (3p26-p24.2, 8q23, and 17q25) previously associated genetically with MMD from Online Mendelian Inheritance in Man (OMIM) [22] and selected antibodies against proteins that were significantly differentially expressed in the serum samples of MMD patients compared to healthy controls at the autoantibody level. Second, we sub-selected for antibodies against proteins that in general were highly differentially expressed across thousands of publicly-available gene expression mRNA microarray experiments, with a fitSNP differential expression ratio (DER) > 0.45. Third, we identified additional proteins that: (a) contained genetic variants associated with any neurological diseases; (b) were significantly differentially expressed in MMD at the autoantibody level; and (c) were highly differentially expressed at the mRNA level with a fitSNP DER > 0.6.

### Validation of expression of MMD-associated autoAbs

Independent sera from a different cohort of MMD patients were used for validation of the autoAbs identified in the ProtoArray discovery phase. The control group of patients in this validation phase consisted of age, gender and demographically-matched patients with other causes of cerebrovascular disease, allowing an assessment of the importance and specificity of the autoAbs in MMD. To validate the significant autoAbs identified by ANOVA and FitSNP analysis, we further selected autoAbs that were known to interact with antigens with biological relevance in vascular and autoimmune disease, where the autoAbs could be detected most easily by ELISA, and where ProtoArray measurements indicated a fold change of > 2 in MMD patients. Customized enzyme-linked immunosorbent assays (ELISAs) were developed as previously described [17] to detect serum immunoglobulin binding to the following array of antigens: amyloid beta A4 protein (APP), catenin, beta 1 (CTNNB1), G protein pathway suppressor 1 (GPS1), stimulated by retinoic acid 13 (STRA13), receptor tyrosine kinase-like orphan receptor 1 (ROR1), and EGF-like repeats and discoidin I-like domains 3 (EDIL3). Briefly, GST-tagged purified proteins (Invitrogen, Carlsbad, CA) were applied to a 96-well immunosorbent plate (NUNC, Rochester, NY) and incubated overnight at 4°C. Standard curves for each protein were prepared using an IgG mouse monoclonal anti-GST tag (Millipore, Temecula, CA) and AP-conjugated AffiniPure goat anti-mouse IgG (Jackson ImmunoResearch, West Grove, PA). The plates were washed with TBST buffer, blocked for 1 hour at room temperature (RT) with 2% dry milk in TBST, and incubated with 50 μL of diluted serum (40-fold with 2% dry milk in TBST buffer) for 1 hour at RT. The plates were then washed with TBST and incubated in AP-conjugated AffiniPure mouse anti-human IgG (Jackson ImmunoResearch, West Grove, PA) and the reactivity was visualized with AP-pNPP Liquid Substrate System for ELISA (Sigma-Aldrich, St. Louis, MO). The signal was measured at 405 nm with a SPECTRAMax 190 microplate reader (Molecular Devices, Sunnyvale, CA). An unpaired t-test p value < 0.05 was considered significant.

## Results

### Identification of MMD-associated autoAbs in the discovery phase by ProtoArray analysis

#### MMD patients express high levels of autoAbs compared to normal healthy controls

**Figure 1:**
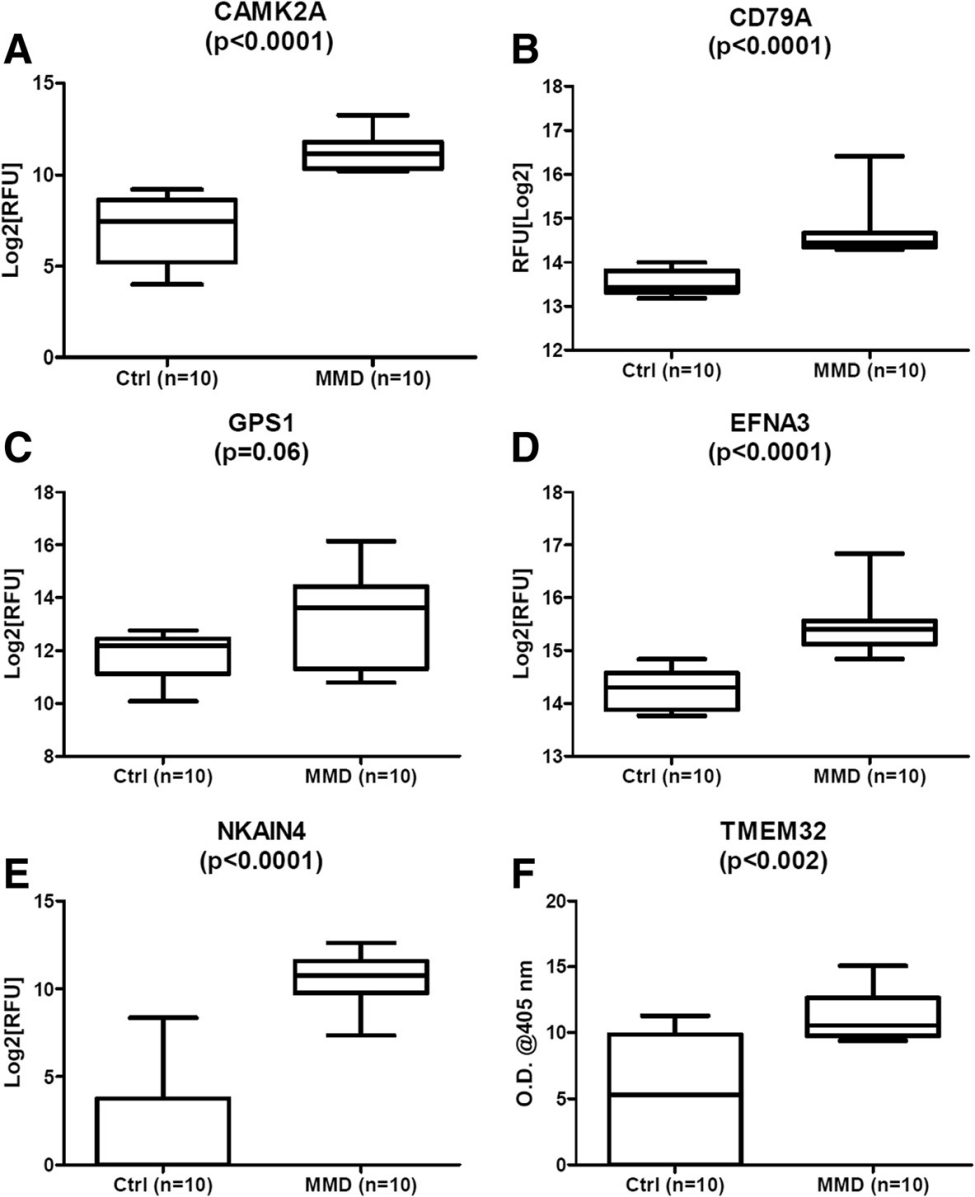
**Increased levels of specific autoAbs were observed in MMD patients compared to healthy controls by protein array.** Targets included (**A**) calcium/calmodulin-dependent protein kinase II alpha (CAMK2A); (**B**) B-cell antigen receptor complex-associated protein alpha-chain (CD79A); (**C**) G protein pathway suppressor 1 (GPS1); (**D**) ephrin-A3 (EFNA3); (**E**) Na+/K + transporting ATPase interacting 4 (NKAIN4); (**F**) Transmembrane Protein 32 (TMEM32). RFU = relative fluorescence units.

In the discovery phase, we evaluated sera samples collected from MMD (n = 10) and healthy normal controls (n = 10) to identify autoAbs significantly associated (p < 0.05) with MMD. A total of 165 autoAbs were identified with a greater than 2-fold change and p ≤0.05 in MMD compared to controls. Table 2 lists 30 of the most significant autoAbs, while the complete list of 165 autoAbs is in Additional file 1: Table S1. Some of the most biologically relevant autoAbs were against antigens with a role in vascular and autoimmune diseases such as: (1) calcium/calmodulin-dependent protein kinase (CaM kinase) II alpha (CAMK2A), which is a signaling molecule implicated in heart failure and arrhythmias and is also known to phosphorylate proteins that are responsible in processes such as excitation–contraction coupling, cell death, and transcriptional activation of hypertrophy and inflammation [23]; (2) calcium/calmodulin-dependent protein kinase ID (CAMK1D) transcript variant 1, which plays a role in regulating calcium-mediated granulocyte function [24]; (3) B-cell antigen receptor complex-associated protein alpha-chain (CD79A), which plays a pivotal role as a B cell antigen receptor complex [25]; (4) EGF-like repeats and discoidin I-like domains 3 (EDIL3), which has a known role in angiogenesis [26]; (5) ephrin-A3 (EFNA3), which is implicated in integrin-mediated T lymphocyte interactions [27] ;(6) CD7, a surface marker for CD8 T cell effector subsets [28]; (7) Na+/K + transporting ATPase interacting 4 (NKAIN4, c20orf58); (8) CDC-like kinase 4 (CLK4), which is a member of CLKs involved in phosphorylation of serine- and arginine-rich (SR) proteins; (9) interferon alpha-inducible protein 6 (IFI6), which is involved in apoptosis [29]; (10) casein kinase 1delta (CSNK1D) transcript variant 1, which is involved in DNA replication and repair [30]; and (11) Type III iodothyronine deiodinase (DIO3), which is involved in thyroid hormone regulation. Figure 1 illustrates measurement levels for 6 autoAbs against the following proteins: CAMK2A, CD79A, G protein pathway suppressor (GPS1), ephrin-A3 (EFNA3), Na+/K + transporting ATPase interacting 4 (NKAIN4), and transmembrane protein 32 (TMEM32).

**Table 2:** Identification of reactive antigens in MMD

**Protein name**	**Gene symbol**	CI* P- value	**Fold increase in MMD**
Calcium/calmodulin-dependent protein kinase (CaM kinase) II alpha (CAMK2A)	CAMK2A	5.41E-06	18.2
B-cell antigen receptor complex-associated protein alpha-chain	CD79A	5.41E-06	2.5
EGF-like repeats and discoidin I-like domains 3 (EDIL3)	EDIL3	5.41E-06	3.5
Ectonucleoside triphosphate diphosphohydrolase 1	ENTPD1	5.41E-06	2.5
Proteinase 3	PRTN3	5.41E-06	2.1
Calcium/calmodulin-dependent protein kinase ID (CAMK1D), transcript variant 1	CAMK1D	5.95E-05	5.1
Ephrin-A3 (EFNA3)	EFNA3	5.95E-05	2.6
CD7 molecule (CD7)	CD7	3.57E-04	2.1
Na+/K + transporting ATPase interacting 4 (NKAIN4)	NKAIN4	3.57E-04	85.2
CDC-like kinase 4 (CLK4)	CLK4	5.47E-04	3.8
Interferon, alpha-inducible protein 6 (IFI6)	IFI6	5.47E-04	2.6
Casein kinase 1, delta (CSNK1D), transcript variant 1	CSNK1D	1.55E-03	5.5
Deiodinase, iodothyronine, type III (DIO3)	DIO3	2.74E-03	5.2
Fc fragment of IgG, low affinity IIIa, receptor (CD16a) (FCGR3A)	FCGR3A	2.74E-03	2.2
RNA Polymerase	RNA POL	2.74E-03	3.7
Uncharacterized protein C20orf96	C20orf96	5.42E-03	4.5
cDNA clone MGC:31944 IMAGE:4878869, complete cds	CD247	5.42E-03	3.0
CD3d molecule, delta (CD3-TCR complex) (CD3D), transcript variant 1	CD3D	5.42E-03	2.8
Endoglin (Osler-Rendu-Weber syndrome 1) (ENG)	ENG	5.42E-03	2.4
Solute carrier family 2 (facilitated glucose transporter), member 2 (SLC2A2)	SLC2A2	5.42E-03	3.7
Serine/threonine kinase 25 (STE20 homolog, yeast) (STK25)	STK25	5.42E-03	2.2
Transmembrane protein 32 (TMEM32)	TMEM32	5.42E-03	10.9
Chromosome 19 open reading frame 39 (C19orf39)	C19orf39	5.42E-03	4.1
Calcium/calmodulin-dependent protein kinase (CaM kinase) II delta (CAMK2D), transcript variant 3	CAMK2D	5.42E-03	3.8
G protein pathway suppressor 1	GPS1	5.42E-03	4.6
Raptor	KIAA1303	5.42E-03	3.0
LPS-responsive vesicle trafficking, beach and anchor containing (LRBA)	LRBA	5.42E-03	3.5
Melanocortin 2 receptor accessory protein (MRAP), transcript variant 1	MRAP	5.42E-03	3.2
NIMA (never in mitosis gene a)-related kinase 3 (NEK3), transcript variant 1	NEK3	5.42E-03	4.3
Receptor (chemosensory) transporter protein 2 (RTP2)	RTP2	5.42E-03	3.4

The following is a subset of the most differentially expressed autoAbs increased by 2 fold or more in MMD compared to healthy controls (p ≤ 0.05).

MMD = moyamoya disease.

* CI p-value = Chebyshev’s Inequality p-value.

Ingenuity Pathway Analysis (IPA) software was applied to all of the reactive antigens and revealed over-represented pathways involved in Neurological Disease, Tissue Development, Cellular Growth and Proliferation, and Muscular System Development and Function. The reactive antigen proteins associated with MMD in the aforementioned pathways were: v-abl Abelson murine leukemia viral oncogene homolog 2 (ABL2), activin A receptor, type IIA (ACVR2A), activin A receptor, type IIA (ACVR2B), CAMK1D, calcium/calmodulin-dependent protein kinase II alpha (CAMK2A), CAMK2D, major histocompatibility complex, class II invariant chain (CD74), CD247, CD3d molecule, delta (CD3D), CSNK1D, EDIL3, E74-like factor 2 (ELF2), endoglin (ENG), ectonucleoside triphosphate diphosphohydrolase 1 (ENTPD1), IL2-inducible T-cell kinase (ITK), kaptin (KPTN), LIM domain kinase 1 (LIMK1), mitogen-activated protein kinase kinase kinase 9 (MAP3K9), myosin light chain kinase 2 (MYLK2), p21 protein (Cdc42/Rac)-activated kinase 4 (PAK4), aspartoacylase (ASPA), beta-site APP-cleaving enzyme 1 (BACE1), DEAD (Asp-Glu-Ala-Asp) box helicase 17 (DDX17), disks large-associated protein DLG7 (DLG7), histone deacetylase 1 (HDAC1), homer homolog 2 (Drosophila) (HOMER2), immunoglobulin (CD79A) binding protein 1 (IGBP1), lipoprotein lipase (LPL), microtubule-associated protein 4 (MAP4), and phospholipid scramblase 1 (PLSCR1). The predominant IPA functions of these reactive antigens were Protein Phosphorylation (p value =1.54E-07) and Protein Modification (p value =1.84E-05), with many of the proteins also involved in pathways deployed in Cell Death (p value =3.74E-05). A panel of autoAbs in MMD was also found to have reactive antigens involved in Cardiac Disease, which is a frequent accompaniment of MMD. The increased autoAb expression against CAMK2D is intriguing, as this protein is implicated in cardiac hypertrophy and heart failure, conditions which have also been reported to coexist with MMD [31]. PRKG1, also antigenic in MMD, has been associated with enlargement of cardiomyocytes and smooth muscle cell proliferation [32,33]. In addition, both LIMK1 and TNFα are associated with cardiac stenosis, while TNFα is specifically associated with cardiac stress response, inflammation, and fibrosis [34-36]. MMD patients exhibited an enrichment of antigenic responses against proteins involved in Immunological Disease and Inflammatory Responses (p value < 2.05E-02), such as AXL receptor tyrosine kinase (AXL), copper metabolism (Murr1) domain containing 1 (COMMD1), DnaJ (Hsp40) homolog, subfamily C, member 12 (DNAJC12) and member 5 beta (DNAJC5B), Fc fragment of IgG, high affinity Ia, receptor (CD64) (FCGR1A) and low affinity IIIa, receptor (CD16a) (FCGR3A), heat shock transcription factor 1 (HSF1), interferon, alpha-inducible protein 27-like 2 (IFI27L2), interferon-induced protein 44-like (IFI44L), interferon, alpha 1/ interferon, alpha 13 (IFNA1/IFNA13), immunoglobulin heavy constant mu (IGHM), immunoglobulin kappa locus (IGK@), interleukin 20 receptor beta (IL20RB), myelin oligodendrocyte glycoprotein (MOG), nuclear factor of kappa light polypeptide gene enhancer in B-cells inhibitor, epsilon (NFKBIE), NFKB repressing factor (NKRF), NLR family, pyrin domain containing 3 (NLRP3), tumor necrosis factor (ligand) superfamily, member 9 (TNFSF9), and ubiquitin-like modifier activating enzyme 7 (UBA7). AutoAbs were also found to be highly enriched against vascular antigens, such as angiotensinogen (AGT), MOG, NLRP3, palmitoyl-protein thioesterase 2 (PPT2), protein kinase C, delta (PRKCD), and SMAD family member 3 (SMAD3).

### A subset of autoAbs is expressed at higher levels during the active disease state as compared to postoperative levels in MMD patients

To determine if there was an acute-MMD autoAb profile, we examined the sera of 5 MMD patients taken prior to surgery and 6 months post-surgery. No significant differences in the majority of MMD-related autoAbs were detected except for 4 autoAbs that were more highly expressed in the sera during active MMD compared to levels 6 months post-surgery. These were Abs against ankyrin repeat and SOCS box-containing 8 (ASB8, p = 0.02), which is known to play a role in intracellular signal transduction [37]; a zinc transporter solute carrier family 39 (zinc transporter) member 9 (SLC39A9, p = 0.02) [38]; and secernin 3 (SCRN3, p = 0.02) and cytochrome P450 family 26 subfamily A polypeptide 1 (CYP26A1, p = 0.02), both of which are expressed in the cerebral cortex [39]. It is unclear why the immune reactivity of this panel of antigens diminishes after surgical correction of the vascular stenosis. There was also a trend, although not significant, of an increase in the postoperative levels of the immunoglobulins IGK@, IGHM, IGHV4-31, and IGKV1-5 (p = 0.08) compared to the active disease state pre-surgery.

### Identification of MMD-associated autoAbs by fitSNP data analysis

Six proteins were selected as significant for MMD, 3 of which were present on MMD-associated loci. The corresponding 6 mRNAs were also highly differentially expressed in publicly-available mRNA microarray experiments, across a variety of diseases with a fitSNP DER > 0.45. Our hypothesis was that these proteins may be involved in the genetic basis of MMD or may be more likely to have SNPs in the translated proteins, driving the immunogenic autoAb response in MMD. We further identified additional proteins that contained genetic variants associated with neurological diseases with cerebrovascular complications that were significantly differentially expressed in MMD at the antibody-level, and that were extremely highly differentially expressed at the mRNA level with a fitSNP DER > 0.6. The following 6 proteins were significantly associated with MMD at the genetic, genomic and antibodyomic level as outlined in Table 3: stimulated by retinoic acid 13 (STRA13), amyloid beta A4 protein (APP), catenin, beta 1 (CTNNB1), G protein pathway suppressor 1 (GPS1), receptor tyrosine kinase-like orphan receptor 1 (ROR1), and EGF-like repeats and discoidin I-like domains 3 (EDIL3).

**Table 3:** Identification of key MMD-associated target genes

**Proteins**	**Enriched p value**	**Function**	**ProtoArray p value**	DER *	**Chromosomal location**
APP	< 0.02	Increased risk of hemorrhagic stroke, Alzheimer disease pathogenesis, DDR [40-42]	0.029	0.604	21q21.3
CTNNB1	0.01	Regulation of neural progenitor cell cycle, Wnt signaling in carcinoma/angiogenesis, chromosome instability [43-45]	0.0197	0.555	3p21
EDIL3	0.01	Role in angiogenesis and vessel wall remodeling and development [46,47]	0.0003	0.484	5q14
ROR1	0.01	Possible role in cell migration and remodeling of cytoskeleton [48]	0.0029	0.586	1p32-p31
GPS1	0.02	Component of the COP9 signalosome, DDR [49]	0.07	0.46	17q25.3
STRA13	0.01	Component of the FA protein complex, DDR, component of the kinetocore [50-52]	0.0021	0.581	17q25.3

MMD-specific target proteins were identified through functional interpolating SNPs approach (fitSNPs), a bioinformatics method that integrates antibodyomic, genetic, and genomic data.

* DER = fitSNP differential expression ratio.

### Validation of potential autoAbs with customized indirect ELISA assays

**Figure 2:**
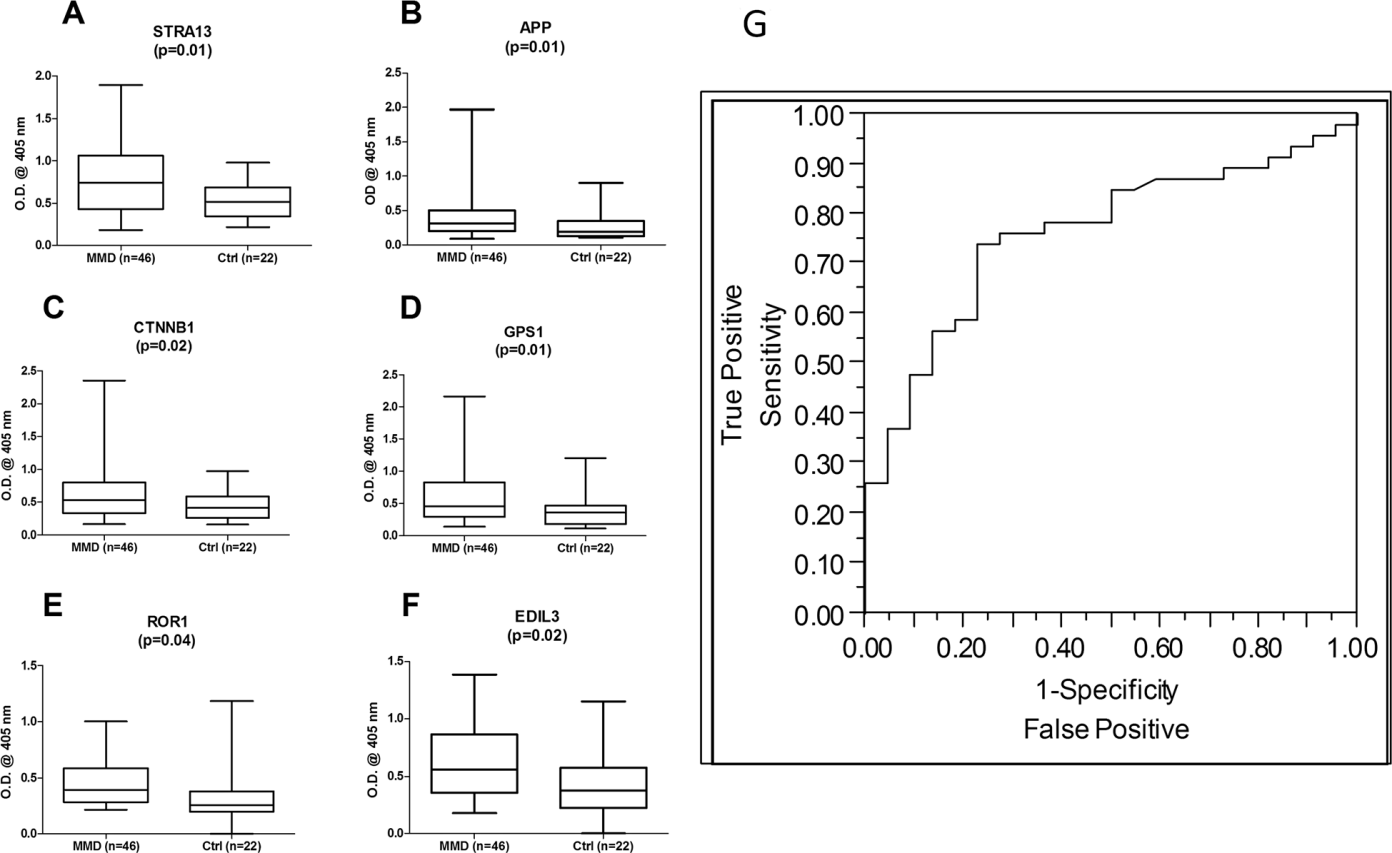
**Validation of autoAb expression by indirect ELISA.** Six MMD-specific proteins identified through a fitSNPs approach were analyzed by indirect ELISA for IgG levels against these antigens in MMD sera. All antigens were found to be significantly increased in MMD (n = 46) compared to CVD controls (n = 22): (**A**) STRA13 (p = 0.01), (**B**) APP (p = 0.01), (**C**) CTNNB1 (p = 0.02), and (**D**) GPS1 (p = 0.01), (**E**) ROR1 (p = 0.04), and (**F**) EDIL3 (p = 0.02) (OD@405 nm = optical density at 405 nm) (**G**) A ROC curve built on a logistic regression model using 6 autoAbs ELISA data (Area Under Curve = 0.76) demonstrates a 74% sensitivity, 77% specificity, 87% PPV, 59% NPV and overall 75% accuracy set of all MMD samples tested.

To further validate the significant presence of these autoAbs identified using the fitSNPs approach, we developed optimized indirect ELISA assays for which reagents were commercially available. ELISA assays were successfully performed on these 6 targets: STRA13, APP, CTNNB1, GPS1, ROR1, and EDIL3. AutoAb expression was examined in a set of independent MMD pre-operative sera (n = 46) compared to cerebrovascular disease controls (n = 22). A significant increase in autoAb levels in MMD was confirmed by reverse ELISA for all 6 targets as shown in Figure 2A-F: STRA13 (p = 0.01), APP (p = 0.01), CTNNB1 (p = 0.02), GPS1 (p = 0.01), ROR1(p = 0.04), EDIL3 (p = 0.02). Statistical analysis of the dataset across gender revealed that the association of AutoAb levels with gender was statistically insignificant except for ROR1 (p = 0.03), indicating the AutoAb levels were associated with the disease phenotype. A logistic regression model was built on the 6 genes from ELISA experimental data (Area Under Curve = 0.76) with 74% sensitivity, 77% specificity, and 87% positive predictive values (PPV) for non-invasive diagnosis of angiographically and histologically confirmed MMD, as shown in Figure 2G.

## Discussion

**Figure 3:**
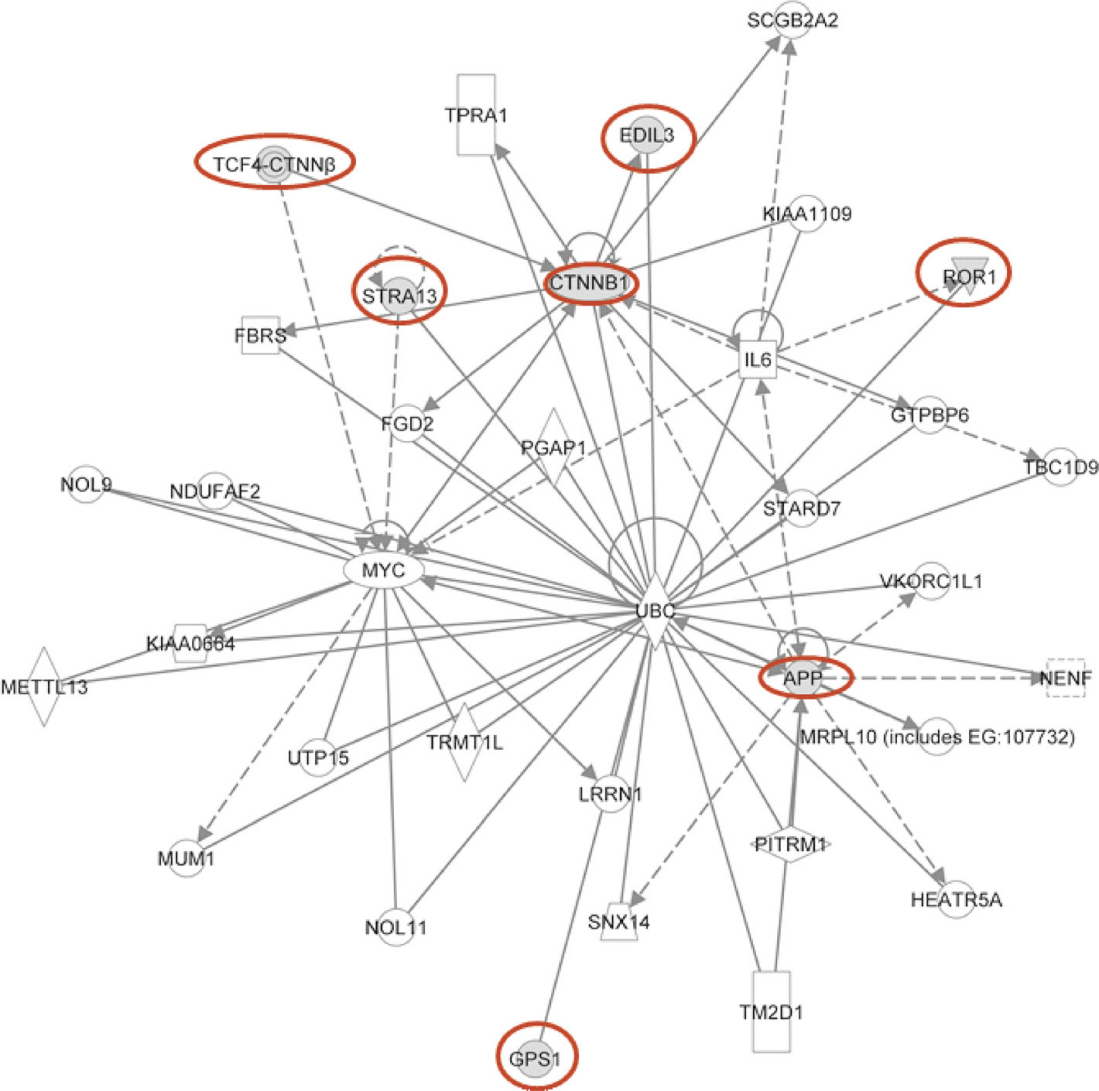
**Association of MMD-specific autoAbs with a Cell-to-Cell Signaling and Interaction, Hematological Disease, and Immunological Disease IPA network.** The autoAbs validated in this study are encircled in bold red.

We report the first comprehensive analysis of the presence of autoAbs in sera of MMD patients. Using high density autoantibody arrays we identified 165 autoAbs associated with MMD as compared to control sera. We further identified a subset of 6 candidate MMD-specific autoAbs, using the novel bioinformatics tool fitSNPs, which were validated by indirect custom-designed ELISAs in a larger cohort of MMD patients (n = 46) compared to CVD control patients (n = 22). These novel autoAbs have not been previously associated with MMD and their precise relationship to MMD remains unknown. The mechanisms leading to autoantibody generation are not yet well described, although several theories exist. These include immune responses to altered protein post-translational modifications, protein mutations and increased levels of protein expression, either as a result of aberrant over-expression or as a consequence of release following cellular reorganization or damage. Ingenuity Pathway analysis, however, revealed that specific pathways were over-represented by the expressed reactive antigens, including Cell-to-Cell Signaling and Interaction, Hematological Disease, and Immunological Disease (Figure 3). These data make biological sense given the nature of MMD, as it is associated with active angiogenesis, smooth muscle cell proliferation and tissue remodeling [1]. However, only 21 autoAbs against antigens with previously described functions in angiogenesis, vascular remodeling/extracellular matrix deposition/, and vascular stability were differentially expressed. These include endoglin (ENG), angiomotin (AMOT) and fibroblast growth factor receptor 1 (FGFR1), in addition to the 4 in the subset of 6 proteins identified by fitSNP (Table 3). Given that MMD is considered a vascular disease, this represents a small percent of the total antigens identified in this paper. There are several possible explanations for this: firstly, the antigens identified in MMD may have as yet undefined roles in angiogenesis, are thus not annotated as such and were not identified in our analysis. This is certainly the case for RNF213, which is currently not present on the array and has only very recently been implicated in MMD and angiogenesis [7]; secondly, the Protein Microarray v4.0 arrays do not contain the full complement of expressed proteins, nor are these angiogenesis-specific arrays; finally, and perhaps most intriguingly, is the fact that these antigens are significantly expressed both pre- and post-surgery. If MMD was a disorder affecting only the cerebrovasculature, we would not only expect more ‘angiogenic’ autoAb expression pre-surgery but for there to be a significant decrease in these same autoAb levels following bypass when the patients have been treated. That autoAb levels remain high both pre- and post-bypass surgery (ie, after the cerebral flow had been re-established) may suggest some novel aspect of the basic disease biology.

Of particular interest are the roles of cell cycle, DNA maintenance and DNA damage response/repair (DDR) pathways. Two highly expressed MMD-associated autoAbs identified in this array with fitSNP and validated by ELISA include those generated against COP9 signalosome complex subunit 1 (GPS1 or CSN1) and stimulated by retinoic acid 13 (STRA13), both of which have well-described functions in DDR. GPS1 is a component of the COP9 signalosome (CSN) complex whose function is to modulate cullin neddylation, which in turn regulates cullin-RING ubiquitin ligase (CRL) activity and the ubiquitination of proteins involved in DDR [49]. The CSN also forms complexes with CRLs containing cullin4 in order to function in DDR, cell cycle regulation and chromatin remodeling [49]. STRA13 (also known as CENP-X or MHF2) is the DNA-binding component of the Fanconi Anemia (FA) core protein complex, through its association with MHF1 and FANCM proteins [50,51]. The FA complex plays a critical role in DDR, and is specifically recruited to stalled DNA replication forks occurring as a result of blockage or damage [53]. Two other validated MMD-specific proteins, Amyloid beta A4 protein (APP) and β-catenin, also have roles in DDR and DNA maintenance. APP, a protein most widely studied for its role in Alzheimer disease, has recently been identified as a component of the TIP60 DNA repair complex, a complex that modifies histone acetylation at DNA double strand breaks [40,41]. β-catenin has a key role in the activation of canonical Wnt signaling and has also recently been implicated in chromosomal instability; specifically, Aoki et al. describe defects in β-catenin leading to activation of Wnt signaling and dysregulation of the cell cycle and chromosomal instability [43]. That DDR and DNA maintenance may have a central role is also supported by the identification in the discovery phase of approximately 20 autoAbs, the antigens against which have known roles in DDR and DNA maintenance, including SWSAP1, CSNK1D, MCM10, NUDT1 and UBE2W.

Further evidence that both MMD/MMS may involve the critical DDR comes from the recent discovery of a novel Xq28 deletion in 3 unrelated hereditary MMS families [6]. This deletion results in the loss of BRCC3, an E3 ubiquitin ligase that is a subunit of the BRCA1-BRCA2-containing complex (BRCC), and deletion of this protein in zebrafish results in defects in angiogenesis [6]. The BRCC complex functions in DDR by stabilizing the BRCA1 protein at DNA break points [54]. Intriguingly, both BRCA1 and BRCA2 are also a part of the FA complex of DDR proteins as described above [53,55]. In addition, MOPD II and Seckel syndrome, diseases strongly associated with moyamoya angiopathy, are sometimes caused by mutations in the pericentrin gene [9,10,56]. Pericentrin is an essential component of the centrosome which mediates, among others, cell cycle progression, spindle organization and orientation, cilia formation and DDR [57].

Given that radiation exposure to the brain is one of the leading environmental causes of moyamoya angiopathy [58], DDR and DNA maintenance machinery may well be compromised in MMD patients. While further research is required to determine if this is the case, precisely how this leads to a complex disease and syndrome remains purely speculative. It is important to note that while defects in DDR, chromosomal instability and cell cycle disregulation often lead to cancer, there is no evidence of increased cancer rates in MMD patients, aside from an association with NF1 [11]. DNA replication and cellular response to DNA damage involve a vast complex of proteins and protein–protein interactions. Given the clinical heterogeneity of MMD and MMS, it would not be unfounded to expect the disease to involve a spectrum of one to several mutations in multiple genes, as is the case with FA patients [53].

The over-representation of antigens pertaining to antigen presentation, immune cell trafficking and inflammatory response may also suggest a significant autoimmune component for MMD. STRA13, one of the validated MMD-specific antigens, also plays an essential role in the stabilization of the outer kinetocore, a protein complex that is essential for correct attachment, segregation and movement of chromosomes during cell division [52]. Reports of the expression of autoAbs to other centromeric proteins of the kinetocore are also associated with a number of autoimmune diseases in humans including rheumatoid arthritis and limited cutaneous sclerosis or CREST syndrome [59]. Therefore, MMD may be related in part to an autoimmune condition.

While the presence of autoAbs may not yet be of diagnostic value in MMD, this prospect may be advanced by validating additional autoAbs and using a larger cohort of patients. Our study demonstrates a novel role for the autoimmune response in MMD and may provide insight into the mechanisms of the disease, including such key cellular processes as cell cycle and DNA repair and maintenance. Future studies are required to further elucidate the clinical relevance of these autoAbs in MMD. There are currently no *in vitro* models of MMD, but recent advances in disease-specific induced pluripotent stem cells (iPSCs) may show some potential as an *in vitro* model of this complex and rare disease.

## Abbreviations

(ACA): Anterior cerebral artery; (autoAbs): Autoantibodies; (CSF): Spinal fluid; (DER): Differential expression ratio; (iPSCs): Induced pluripotent stem cells; (ICA): Internal carotid artery; (MCA): Middle cerebral artery; (MMD): Moyamoya disease; (MMS): Moyamoya syndrome; (TIA): Transient ischemic attack.

## Competing interests

The authors indicate there are no competing interests.

## Authors’ contributions

TKS performed the autoAb arrays, analyzed the data and assisted in manuscript preparation, LDS drafted the manuscript, RC assisted in analyzing the data and in manuscript preparation, LL assisted in analyzing the data, AJB assisted in analyzing the data, MMS and GKS conceived the project and participated in study design and in manuscript preparation. All authors have read and approved the final manuscript.

## Authors’ information

Minnie M Sarwal and Gary K Steinberg are Joint Senior Authors.

## Supplementary Material

Additional file 1: Table S1 List of reactive antigens indentified in MMD sera. The following 165 autoAbs were significantly over-expressed in MMD compared to healthy controls (p≤0.05). Click here for file
