# Supplementary material for: Immune response profiling identifies autoantibodies specific to Moyamoya patients
Source: Orphanet J Rare Dis. 2013 Mar 21;8:45. doi: 10.1186/1750-1172-8-45 (PMC3648437; doi:10.1186/1750-1172-8-45)
Supplement: Additional file 1: Table S1 — List of reactive antigens indentified in MMD sera. The following 165 autoAbs were significantly over-expressed in MMD compared to healthy controls (p≤0.05). [file 1750-1172-8-45-S1.doc]

**Supplementary Information**

**Supplementary Table 1.** List of reactive antigens indentified in MMD sera. The following 165 autoAbs were significantly over-expressed in MMD compared to healthy controls (p≤0.05).

| **S. No.** | **Refseq_ID/Accession no.** | **Gene Symbol** | **Mean RFU Ctrl** | **Mean RFU MMD** | **P-Value** |
| --- | --- | --- | --- | --- | --- |
| 1 | NM_212492.1 | GPS1 | 3830.9 | 17448.6 | 5.42E-03 |
| 2 | NP_741960 | CAMK2A | 163.3 | 2973.9 | 5.41E-06 |
| 3 | BC041037.1 | IGHM | 5140.5 | 18557.3 | 5.41E-06 |
| 4 | BC053656.1 | EDIL3 | 11540.0 | 40401.3 | 5.41E-06 |
| 5 | BC056256.1 | IGKC | 10627.5 | 37094.5 | 5.41E-06 |
| 6 | BC053984.1 | IGHV4-31 | 24879.3 | 69251.9 | 5.41E-06 |
| 7 | BC005332.1 | ENTPD1 | 24662.4 | 61999.8 | 5.41E-06 |
| 8 | BC022362.1 | CD79A | 10869.3 | 26854.3 | 5.41E-06 |
| 9 | BC025314.1 | IGHG1 | 25811.1 | 59136.4 | 5.41E-06 |
| 10 | BC034146.1 | IGKV1-5 | 16870.0 | 37851.5 | 5.41E-06 |
| 11 | BC016381.1 | IGHM | 29437.8 | 63371.4 | 5.41E-06 |
| 12 | NC_000019.9 | PRTN3 | 5038.5 | 10360.3 | 5.41E-06 |
| 13 | NP_705718 | CAMK1D | 469.8 | 2404.9 | 5.95E-05 |
| 14 | BC030814.1 | IGKV1-5 | 11778.7 | 43713.3 | 5.95E-05 |
| 15 | BC034142.1 | IGKV1-5 | 9338.5 | 27157.4 | 5.95E-05 |
| 16 | BC034141.1 | IGKC | 9538.6 | 25448.3 | 5.95E-05 |
| 17 | NM_004952.3 | EFNA3 | 17630.3 | 45778.9 | 5.95E-05 |
| 18 | BC029444.1 | IGKC | 12010.1 | 30913.8 | 5.95E-05 |
| 19 | BC016380.1 | IGK@ | 13347.0 | 34115.0 | 5.95E-05 |
| 20 | BC033178.1 | IGHG3 | 11245.4 | 28605.4 | 5.95E-05 |
| 21 | BC032451.1 | IGK@ | 17796.2 | 45227.2 | 5.95E-05 |
| 22 | NM_152864.2 | C20orf58 | 27.3 | 2322.0 | 3.57E-04 |
| 23 | BC032452.1 | IGL@ | 9049.8 | 25761.6 | 3.57E-04 |
| 24 | BC030983.1 | IGL@ | 20598.8 | 54258.6 | 3.57E-04 |
| 25 | BC030984.1 | CD7 | 19971.2 | 42229.9 | 3.57E-04 |
| 26 | NP_065717 | CLK4 | 1657.9 | 6266.5 | 5.47E-04 |
| 27 | BC024289.1 | IFI6 | 8418.8 | 21651.0 | 5.47E-04 |
| 28 | NP_620693 | CSNK1D | 411.8 | 2282.9 | 1.55E-03 |
| 29 | BC017717.1 | DIO3 | 881.7 | 4576.2 | 2.74E-03 |
| 30 | BC017865.1 | FCGR3A | 6002.5 | 13145.8 | 2.74E-03 |
| 31 | NM_173470.1 | TMEM32 | 526.2 | 5746.0 | 5.42E-03 |
| 32 | BC051762.1 | C20orf96 | 2802.0 | 12624.8 | 5.42E-03 |
| 33 | BC060041.1 | SLC2A2 | 3631.1 | 13428.4 | 5.42E-03 |
| 34 | BC054893.1 | IGLV2-14 | 4285.7 | 13783.1 | 5.42E-03 |
| 35 | BC022098.1 | CD247 | 3625.3 | 10947.3 | 5.42E-03 |
| 36 | BC015833.1 | CD3D | 5233.6 | 14737.6 | 5.42E-03 |
| 37 | BC014271.2 | ENG | 3504.1 | 8400.0 | 5.42E-03 |
| 38 | NP_006365 | STK25 | 2495.9 | 5568.5 | 5.42E-03 |
| 39 | BC003400.1 | TAF9 | 746.2 | 15442.1 | 5.42E-03 |
| 40 | NM_053067.1 | UBQLN1 | 3534.2 | 16205.6 | 5.42E-03 |
| 41 | NP_689933 | NEK3 | 1660.0 | 7145.4 | 5.42E-03 |
| 42 | NM_004906.3 | WTAP | 4125.4 | 17482.1 | 5.42E-03 |
| 43 | NM_147197.2 | WFDC11 | 3536.3 | 14817.6 | 5.42E-03 |
| 44 | BC068071.1 | C19orf39 | 3710.6 | 15230.0 | 5.42E-03 |
| 45 | NP_742113 | CAMK2D | 909.8 | 3446.3 | 5.42E-03 |
| 46 | BC063107.1 | TPO | 3655.2 | 13325.6 | 5.42E-03 |
| 47 | BC064834.1 | LRBA | 3017.7 | 10683.3 | 5.42E-03 |
| 48 | NM_006552.1 | SCGB1D1 | 4275.8 | 14643.7 | 5.42E-03 |
| 49 | NM_001004312.1 | RTP2 | 4226.2 | 14404.0 | 5.42E-03 |
| 50 | NM_178817.3 | MRAP | 3620.5 | 11523.3 | 5.42E-03 |
| 51 | BC064515.1 | KIAA1303 | 4799.3 | 14478.6 | 5.42E-03 |
| 52 | NM_013444.2 | UBQLN2 | 3597.9 | 10533.6 | 5.42E-03 |
| 53 | NP_009298.1 | ABL2 | 2685.6 | 6711.1 | 9.88E-03 |
| 54 | BC033537.1 | KIAA1576 | 4778.0 | 26705.6 | 1.63E-02 |
| 55 | BC051031.1 | C11orf74 | 5496.5 | 25260.6 | 1.63E-02 |
| 56 | BC054034.1 | U1SNRNPBP | 2963.9 | 12779.5 | 1.63E-02 |
| 57 | BC060824.1 | ARID3B | 3060.2 | 12310.7 | 1.63E-02 |
| 58 | NM_133265.2 | AMOT | 6792.5 | 27132.0 | 1.63E-02 |
| 59 | NM_032017.1 | STK40 | 2069.0 | 7542.9 | 1.63E-02 |
| 60 | NM_198949.1 | NUDT1 | 3810.1 | 12314.7 | 1.63E-02 |
| 61 | NM_207365.1 | AADACL2 | 3778.6 | 12053.0 | 1.63E-02 |
| 62 | BC036723.1 | FCGR3A | 5012.5 | 15552.9 | 1.63E-02 |
| 63 | BC070189.1 | LOC541473 | 3889.6 | 11798.4 | 1.63E-02 |
| 64 | NM_001106.2 | ACVR2B | 5706.1 | 17279.1 | 1.63E-02 |
| 65 | BC069185.1 | FCRL2 | 4715.2 | 14056.0 | 1.63E-02 |
| 66 | NM_002307.1 | LGALS7 | 4984.1 | 13913.4 | 1.63E-02 |
| 67 | NM_004964.2 | HDAC1 | 4788.3 | 13264.7 | 1.63E-02 |
| 68 | BC012109.1 | HOMER2 | 8534.1 | 22482.2 | 1.63E-02 |
| 69 | NP_005537 | ITK | 733.4 | 1871.4 | 1.63E-02 |
| 70 | NM_002618.2 | PEX13 | 4274.3 | 10688.8 | 1.63E-02 |
| 71 | NM_145716.2 | SSBP3 | 2380.1 | 14888.9 | 2.86E-02 |
| 72 | NM_018070.2 | SSBP3 | 3237.4 | 18889.2 | 2.86E-02 |
| 73 | NM_002254.5 | KIF3C | 2387.4 | 12646.9 | 2.86E-02 |
| 74 | NM_017897.1 | OXSM | 4344.8 | 20661.1 | 2.86E-02 |
| 75 | NM_002946.2 | RPA2 | 2649.0 | 12450.1 | 2.86E-02 |
| 76 | BC017936.1 | GAMT | 5529.1 | 21490.1 | 2.86E-02 |
| 77 | BC009485.1 | C4orf16 | 3360.0 | 12930.5 | 2.86E-02 |
| 78 | NM_033506.1 | FBXO24 | 1797.5 | 6665.1 | 2.86E-02 |
| 79 | NP_149109 | MYLK2 | 1977.6 | 7060.1 | 2.86E-02 |
| 80 | NP_003168 | SYK | 538.8 | 1426.3 | 2.86E-02 |
| 81 | NM_203284.1 | RBPJ | 10745.8 | 23221.5 | 2.86E-02 |
| 82 | NM_138972.2 | BACE1 | 3233.7 | 16310.7 | 3.49E-02 |
| 83 | NM_003782.3 | B3GALT4 | 1749.2 | 8006.6 | 3.49E-02 |
| 84 | BC029541.1 | LETM2 | 2059.2 | 9359.7 | 3.49E-02 |
| 85 | BC011399.1 | SYK | 4248.8 | 18759.1 | 3.49E-02 |
| 86 | BC051843.1 | MAP4 | 3342.9 | 13143.3 | 3.49E-02 |
| 87 | BC026175.1 | ATF2 | 4096.9 | 15859.8 | 3.49E-02 |
| 88 | NM_001616.2 | ACVR2A | 2838.1 | 9935.7 | 3.49E-02 |
| 89 | BC000468.1 | UBE2V1 | 7568.2 | 19568.3 | 3.49E-02 |
| 90 | NM_001006634.1 | ARHGAP17 | 2871.6 | 16429.1 | 4.33E-02 |
| 91 | BC030608.2 | PODN | 3988.2 | 20640.4 | 4.33E-02 |
| 92 | BC004259.1 | C14orf140 | 3179.0 | 15059.2 | 4.33E-02 |
| 93 | NM_053285.1 | TEKT1 | 2161.7 | 9351.0 | 4.33E-02 |
| 94 | BC051849.2 | RPAIN | 2902.5 | 11710.2 | 4.33E-02 |
| 95 | BC013019.1 | CCDC28A | 2839.1 | 10683.6 | 4.33E-02 |
| 96 | NM_181705.1 | LYRM7 | 4001.9 | 14633.6 | 4.33E-02 |
| 97 | NM_001018116.1 | LOC347273 | 1910.0 | 6485.4 | 4.33E-02 |
| 98 | BC004106.1 | MED6 | 3312.7 | 10244.0 | 4.33E-02 |
| 99 | NM_016735.1 | LIMK1 | 2715.2 | 7759.0 | 4.33E-02 |
| 100 | NM_016207.2 | CPSF3 | 3195.9 | 8497.8 | 4.33E-02 |
| 101 | NM_032360.1 | ACBD6 | 3957.6 | 9498.0 | 4.33E-02 |
| 102 | BC015219.1 | C20orf18 | 4203.9 | 9298.2 | 4.33E-02 |
| 103 | BC010887.1 | REC8 | 2280.8 | 15834.0 | 4.33E-02 |
| 104 | NM_002436.2 | MPP1 | 2247.3 | 14005.6 | 4.33E-02 |
| 105 | NM_024046.1 | CAMKV | 1710.1 | 10486.9 | 4.33E-02 |
| 106 | BC056160.1 | SLC25A5 | 2118.4 | 12826.9 | 4.33E-02 |
| 107 | BC009877.1 | P2RY11 | 1704.3 | 10308.8 | 4.33E-02 |
| 108 | BC005153.1 | RPH3AL | 1908.9 | 11103.9 | 4.33E-02 |
| 109 | BC016276.1 | DLG7 | 2246.8 | 12455.9 | 4.33E-02 |
| 110 | NM_014286.2 | FREQ | 1848.9 | 10117.4 | 4.33E-02 |
| 111 | NM_018107.2 | RBM23 | 2194.8 | 11948.6 | 4.33E-02 |
| 112 | NM_001997.2 | FAU | 1775.3 | 9559.9 | 4.33E-02 |
| 113 | BC004876.1 | MCM10 | 2266.3 | 12013.9 | 4.33E-02 |
| 114 | BC022988.1 | C6orf65 | 2110.7 | 10976.1 | 4.33E-02 |
| 115 | NM_016564.1 | CEND1 | 1999.7 | 10373.5 | 4.33E-02 |
| 116 | NM_004422.1 | DVL2 | 2027.8 | 10454.4 | 4.33E-02 |
| 117 | BC017066.1 | PRRC1 | 2037.2 | 10464.7 | 4.33E-02 |
| 118 | NM_004922.2 | SEC24C | 2781.5 | 14210.3 | 4.33E-02 |
| 119 | BC062688.1 | PRKG1 | 2356.2 | 11982.4 | 4.33E-02 |
| 120 | NM_004632.2 | DAP3 | 2009.5 | 10162.8 | 4.33E-02 |
| 121 | BC000001.1 | CHID1 | 2139.2 | 10698.4 | 4.33E-02 |
| 122 | NM_021809.2 | TGIF2 | 1970.6 | 9816.8 | 4.33E-02 |
| 123 | BC028983.1 | FXR1 | 2396.7 | 11922.0 | 4.33E-02 |
| 124 | NM_020317.2 | C1orf63 | 2931.7 | 14491.2 | 4.33E-02 |
| 125 | BC011353.1 | LPL | 2389.2 | 11641.6 | 4.33E-02 |
| 126 | BC009398.1 | MCM7 | 1356.1 | 6577.3 | 4.33E-02 |
| 127 | NP_000595 | FGFR1 | 740.4 | 3570.9 | 4.33E-02 |
| 128 | NM_201999.1 | ELF2 | 2591.5 | 12043.0 | 4.33E-02 |
| 129 | BC001946.1 | BRUNOL4 | 2238.6 | 10387.8 | 4.33E-02 |
| 130 | BC024272.1 | CD74 | 2212.5 | 10125.3 | 4.33E-02 |
| 131 | NM_000284.1 | PDHA1 | 2532.2 | 11563.9 | 4.33E-02 |
| 132 | NM_002784.2 | PSG9 | 1961.5 | 8897.2 | 4.33E-02 |
| 133 | BC000238.1 | ANKZF1 | 2605.1 | 11753.3 | 4.33E-02 |
| 134 | NM_014267.2 | C11orf58 | 2091.4 | 9022.8 | 4.33E-02 |
| 135 | NM_032324.1 | C1orf57 | 2950.7 | 12575.0 | 4.33E-02 |
| 136 | BC018928.1 | SEC24C | 3106.1 | 12843.7 | 4.33E-02 |
| 137 | NM_002708.1 | PPP1CA | 2181.5 | 9017.3 | 4.33E-02 |
| 138 | NM_000594.2 | TNF | 2954.2 | 12000.5 | 4.33E-02 |
| 139 | BC006318.1 | EPB49 | 1704.1 | 6904.6 | 4.33E-02 |
| 140 | NM_016630.2 | SPG21 | 2032.5 | 8102.0 | 4.33E-02 |

| 141 | NM_005513.1 | GTF2E1 | 3395.2 | 12287.7 | 4.33E-02 |
| --- | --- | --- | --- | --- | --- |
| 142 | NM_024567.2 | HMBOX1 | 3966.4 | 14314.9 | 4.33E-02 |
| 143 | NM_013368.2 | SERTAD3 | 2208.4 | 7635.6 | 4.33E-02 |
| 144 | NM_178832.2 | C10orf83 | 2857.9 | 9724.4 | 4.33E-02 |
| 145 | NM_207005.1 | USF1 | 4321.4 | 14516.6 | 4.33E-02 |
| 146 | BC009249.1 | KPTN | 1986.3 | 6508.0 | 4.33E-02 |
| 147 | BC038953.1 | COG3 | 1399.5 | 4530.0 | 4.33E-02 |
| 148 | NM_005371.2 | METTL1 | 3575.3 | 11488.3 | 4.33E-02 |
| 149 | BC052805.1 | EPB49 | 7841.0 | 24439.2 | 4.33E-02 |
| 150 | BC012609.1 | SERPINB2 | 2724.8 | 8463.3 | 4.33E-02 |
| 151 | NM_015963.4 | THAP4 | 3938.0 | 12163.9 | 4.33E-02 |
| 152 | NM_002931.3 | RING1 | 4973.9 | 15104.3 | 4.33E-02 |
| 153 | BC013178.1 | SFRS16 | 4411.3 | 12733.9 | 4.33E-02 |
| 154 | NM_001007246.1 | BRWD1 | 2745.5 | 7873.9 | 4.33E-02 |
| 155 | NM_032641.1 | SPSB2 | 4438.0 | 12100.5 | 4.33E-02 |
| 156 | NM_001551.1 | IGBP1 | 5226.9 | 13928.4 | 4.33E-02 |
| 157 | NM_002629.2 | PGAM1 | 3265.8 | 8502.7 | 4.33E-02 |
| 158 | NM_000723.3 | CACNB1 | 4029.9 | 10456.4 | 4.33E-02 |
| 159 | NM_021105.1 | PLSCR1 | 4834.0 | 11839.7 | 4.33E-02 |
| 160 | NM_000049.1 | ASPA | 2139.4 | 5028.5 | 4.33E-02 |
| 161 | NM_178151.1 | DCX | 4208.6 | 9776.7 | 4.33E-02 |
| 162 | NM_002774.2 | KLK6 | 5184.2 | 12033.5 | 4.33E-02 |
| 163 | NP_001996 | FES | 797.7 | 1836.5 | 4.33E-02 |
| 164 | BC012997.2 | SULF1 | 10040.5 | 22501.1 | 4.33E-02 |
| 165 | NM_000266.1 | NDP | 2595.8 | 10250.3 | 5.42E-03 |
